# A rare case of unusual anti-tuberculosis drug reaction in disseminated tuberculosis with pre-existing Multisystem Inflammatory Syndrome in Children (MIS-C)

**DOI:** 10.1186/s12879-026-12514-4

**Published:** 2026-01-09

**Authors:** Riyadi Adrizain, Dyah Ayu Shinta Lesmanawati, Fadila Dyah Trie Utami, Anggraini Alam, Djatnika Setiabudi

**Affiliations:** 1 Department of Child Health, Faculty of Medicine, Universitas Padjadjaran/Hasan Sadikin General Hospital, Bandung, West Java Indonesia; 2 Pediatric Resident, Department of Child Health, Faculty of Medicine, Universitas Padjadjaran/Hasan Sadikin General Hospital, Bandung, West Java Indonesia

**Keywords:** Multisystem inflammatory syndrome in children, Tuberculosis, Case report, Drug eruption

## Abstract

Children with *Multisystem Inflammatory Syndrome in Children (MIS-C)* often have weakened immune systems and more prone to secondary infections such as tuberculosis (TB). *MIS-C* is a rare case but serious complication of COVID-19. It commonly affects the gastrointestinal, cardiovascular, hematologic, and mucocutaneous systems. This study presents a case of MIS-C in a 15-year-old girl complicated by respiratory failure, increased intracranial pressure with status epilepticus, grade II TB meningitis, severe dengue, disseminated TB, anti-tuberculosis drug induced hepatotoxicity, and cutaneous drug eruption. The patient had a history of prior COVID-19 infections and arrived the emergency department with high fever, loss of consciousness, seizure, a generalized maculopapular rash, and respiratory failure. Laboratory results showed significantly elevated liver enzyme, while chest X-ray confirmed pleural effusion and pulmonary TB. A multidisciplinary team was formed, including specialists in respirology, cardiology, pediatric neurology, immunology, and gastro-hepatology division. The patient received high dose of steroid, anti-tuberculosis regiment, and phenobarbital to control the seizure. N-acetylcysteine and enoxaparin were administered as a part of the MIS-C treatment protocol. The patient was discharged after three weeks of intensive care but required monthly readmission due to recurrent complications. This study suggests the importance of early detection and comprehensive management of MIS-C to reduce the risk of mortality and secondary infection in long-term impact of the syndrome.

## Background

Tuberculosis (TB) become a major public health challenge in Indonesia. Pediatric populations remain particularly vulnerable to TB and Indonesia ranked second worldwide for TB prevalence [1]. The most common adverse drug reactions during TB treatment involve severe or life – threatening cases that may require treatment adjustment or discontinuation. These reactions can also lead to longer hospital stays and more severe illness [2]. 

According to the World Health Organization (WHO), more than 775 million confirmed cases of COVID-19 had been recorded worldwide by April 2024, and 7 million of them were deaths [3]. In Indonesia, the incidence of the pandemic is high, 13,8% of cases were recorded in population under 18 years [4]. The mortality rate of 0–5 and 6–18 age group is 0.9 and 1.8%, respectively [5]. This showed the significant impact of COVID-19 among children in Indonesia. Some patients showed symptoms like *toxic shock syndrome*, but microbial infection was not confirmed. These patients looked very ill with multi organ damage, which is not typical in pediatric COVID-19 cases [6]. The WHO defined this condition as Multisystem Inflammatory Syndrome in Children (MIS-C) [7, 8]. To facilitate early diagnosis of MIS-C, the Centers for Disease Control and Prevention (CDC) and WHO have established clinical and laboratory criteria for a life-threatening condition that often requires intensive care and mechanical ventilation [9]. Driven by an excessive immune response, the syndrome develops as a post-infectious complication rather than a manifestation of the acute infection. MIS-C is a rare complication which occurs in less than 1% of pediatrics with very few cases reported in Asia [10]. Indonesia is being heavily impacted by the COVID-19 pandemic, but the data of this syndrome remains limited [11]. The diagnosis and management are often delayed due to limited knowledge and clinical experience. Lack of family education may delay prompt medical attention [12]. Additionally, limited access to healthcare services, the high cost of diagnostic procedures, and limited availability of immunomodulatory therapies can also worsen outcomes in MIS-C cases [13]. Children with MIS-C may experience a severe inflammatory condition that causes significant damage to vital organs such as nervous system, cardiovascular system, and neurological system, leading to chronic conditions or death [14]. 

This study reports an unusual drug reaction in a patient diagnosed with MIS-C and complicated by disseminated tuberculosis (TB). The use of corticosteroids in MIS-C patients may worsen infections. Glucocorticoid therapy increases the risk of developing new-onset tuberculosis, and moderate to high doses are associated with reactivation of latent TB. This occurs because suppression of the pro-inflammatory cytokine production and impairing macrophage phagocytic activity, leading to a down-regulation of immune system, These contribute to a higher risk of tuberculosis reactivation [15, 16]. In addition, the Immunosuppression in MIS-C patients itself increases vulnerability to secondary infections such as TB, CMV or EBV which can reactivate these pathogens and spread to organs such as the liver, kidneys, brain, and bones, leading to severe complications [10, 17]. Early detection and comprehensive management are crucial to prevent severe outcomes.

## Case presentation

**Fig. 1:**
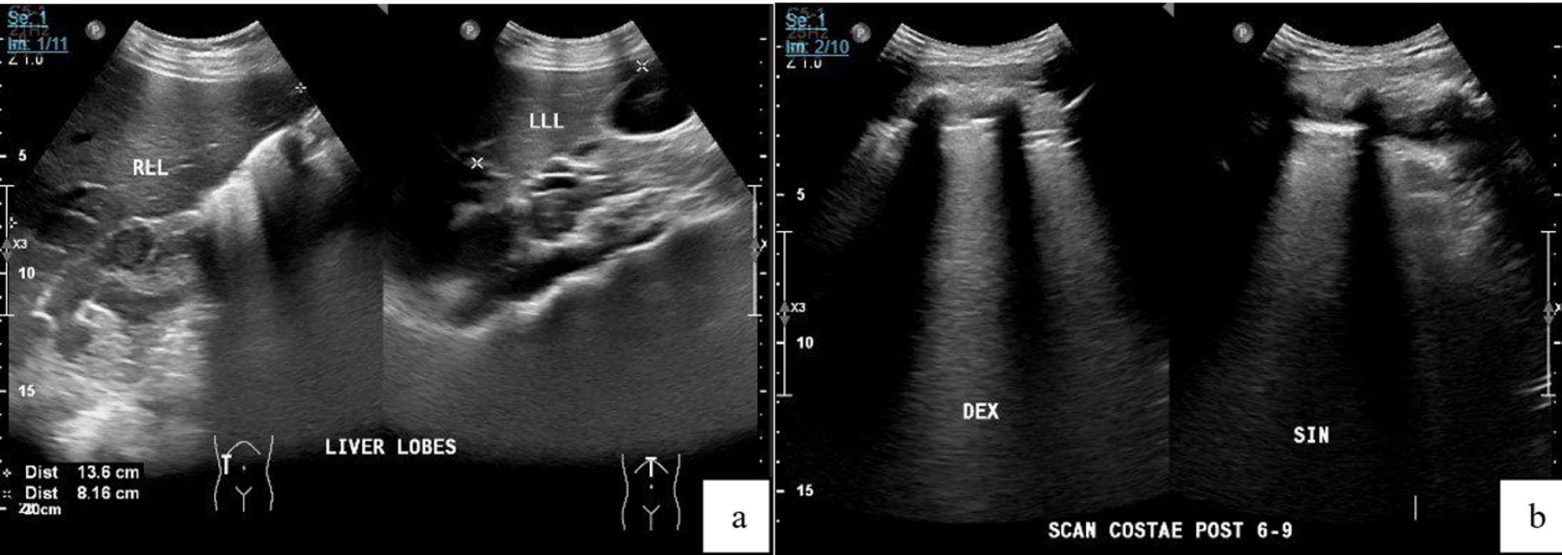
Ultrasonography of the patient (**a**) Abdomen and (**b**) Thorax

A 15-year-old girl was come to the emergency department in November 2023 with loss of consciousness and drowsiness, following two days of persistent nausea and vomiting. She also had high fever and headache for five days before admission. The vomiting was frequent, containing yellowish fluid but no blood. Over the previous two months, she had lost 8 kg of weight. There was a history of COVID-19 infection one month before and only required home isolation due to mild symptoms. No history of TB treatment, nor a family history of TB, autoimmune disease, asthma, or allergies was recorded, but the father had a smoking history. The patient immunizations were reportedly complete, including two doses of the COVID-19 vaccine, and there was no BCG vaccination scar. During observation, she developed seizures and respiratory failure, requiring mechanical ventilation and Pediatric Intensive Care Unit (PICU) admission. A generalized maculopapular rash was noted, along with cold extremities, hypotension, and bradycardia. Laboratory results identified leucocytosis, thrombocytopenia, prolonged PT/aPTT/INR, elevated D-dimer, CRP, and procalcitonin. Liver enzymes were markly elevated (SGOT/SGPT: 2007/943), and CKMB levels were also increased as detailed in Table 1. Dengue IgG was reactive. Ultrasonography of abdomen was performed, and the results showed no abnormality, while ultrasonography of thorax presented anechoic lesion with septate in the right supradiaphragmatic area, consistent with septate pleural effusion, as detailed in Fig. 1.

**Table 1:** Supporting examination resultss

Time Course	November 2023	December 2024	January 2024
Laboratory Result	Hb: 13.9 Ht: 40.4 Erythrocyte: 4.95 Leukocyte: 16.080 Platelet: 122.000 Na: 123 K:3.4 Cl: 90 CRP: 26.59 Procalcitonin 9.91 SGOT/SGPT; 2007/943 Total Bilirubin: 2.397, Direct Bilirubin 0.830, Indirect Bilirubin: 1.567 Lactic acid: 3.1 D-dimer 11.29 PT/APTT/INR: 19.5/28.10/1.45 Ur/Cr: 13.6/0.47 Anti-HIV non-reactive, ANA Test: non-reactive. Anti-Dengue IgG reactive, IgM Non-reactive	Hb: 11.8 Ht: 37.7 Erythrocyte: 4.23 Leukocyte: 4.370 Platelet: 172.000 Na: 136 K:2.9 Cl: 99 CRP: 1.19 Procalcitonin 3.32 SGOT/SGPT: 66/244 Total Bilirubin: 0.795, Direct Bilirubin 0.385, Indirect Bilirubin: 0.410 Lactic acid: 2 D-dimer 2.89 PT/APTT/INR: 13.3/32.6/0.95 HbsAg: non-reactive, Anti HCV: non-reactive	Hb:11.4 Ht: 32.9 Erythrocyte: 3.99 Leukocyte: 1.520 Platelet: 347.000 CRP: 4.23 SGOT/SGPT: 99/157 Total Bilirubin: 0.704, Direct Bilirubin: 0.404, Indirect Bilirubin: 0.300 Ur/Cr: 122/0.6 HbsAg: non-reactive, Anti HCV non-reactive
Blood Gas Analysis	pH: 7.420 pCO2: 32 pO2: 83.3 HCO3: 21.1 tCO2: 22.1 Be-b: -1.9 SpO2: 96.3	pH: 7.470 pCO2: 38.4 pO2: 129.2 HCO3: 28.4 tCO2: 29.5 Be-b: 5.1 SpO2: 98.6	
AFB Sputum	**-/-**	**+/+**	
TCM	**-**	Sputum: Rif Sensitive Fesses: Rif Sensitive Urine: Indetermined	
Culture	No Organism	E-coli in urine sample	No Organism in blood
USG	Thorax: Anechoic lesion with septate in right supradiaphragmatic suggested effusion pleura Abdominal: No Abnormality		
Chest X-ray		Right Pleural Effusion	No Pleural Effusion

The patient was diagnosed for MIS-C, complicated by respiratory failure, elevated intracranial pressure with status epilepticus, suspected grade II tuberculous meningitis, and severe dengue (including dengue shock syndrome and dengue encephalopathy). In the canter, all the diagnostic test to fulfil the criteria of the syndrome were available. A multidisciplinary team was formed, including pediatric respirology, neurology, cardiology, gastro-hepatology, and immunology. The patient received high-dose intravenous corticosteroids. Levofloxacin (750 mg) and amikacin (750 mg once daily) were administered as part of the anti-tuberculosis regimen because only parenteral formulations were available, and the patient’s unconscious condition required intravenous administration. Seizures were managed with phenobarbital, and MIS-C-specific treatment included 600 mg N-acetylcysteine and 30 mg enoxaparin twice daily. Following three weeks of hospitalization and intensive care, the patient was discharged in stable condition and good prognosis.

**Fig. 2:**
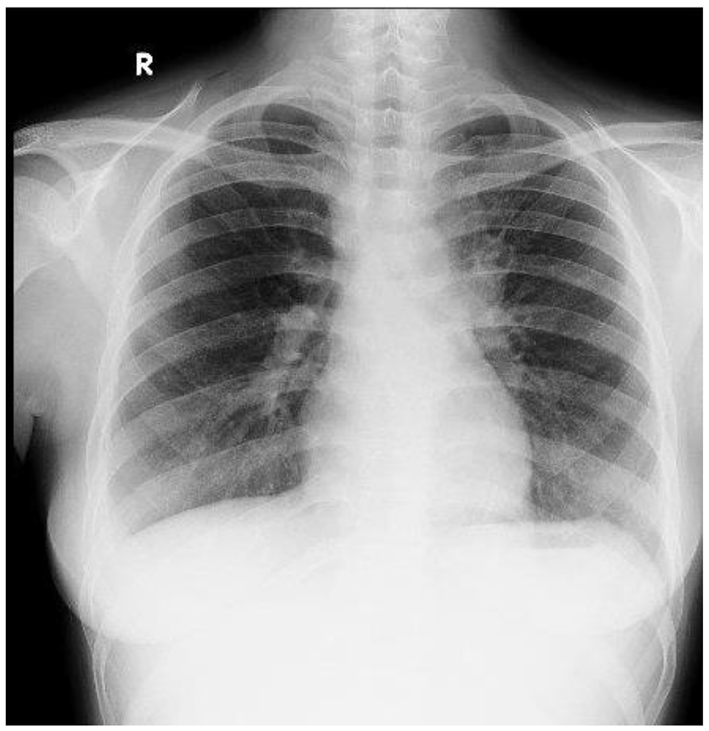
Chest X-Ray of the patient

Two weeks after discharge, the patient returned to the emergency department with shortness of breath and fever for one week. Oxygen saturation was 89% on room air, and right submandibular lymphadenopathy was observed. A chest X-ray showed miliary pulmonary TB with right-sided pleural effusion, as detailed in Fig. 2. Further evaluation identified TB bacteria in stool, urine, and lymph nodes, leading to a diagnosis of disseminated TB. Anti-TB treatment was continued using a fixed-dose combination (FDC) pediatric regimen (1 × 4 tablets/day) and oral prednisone (5 mg, three times daily).

**Fig. 3:**
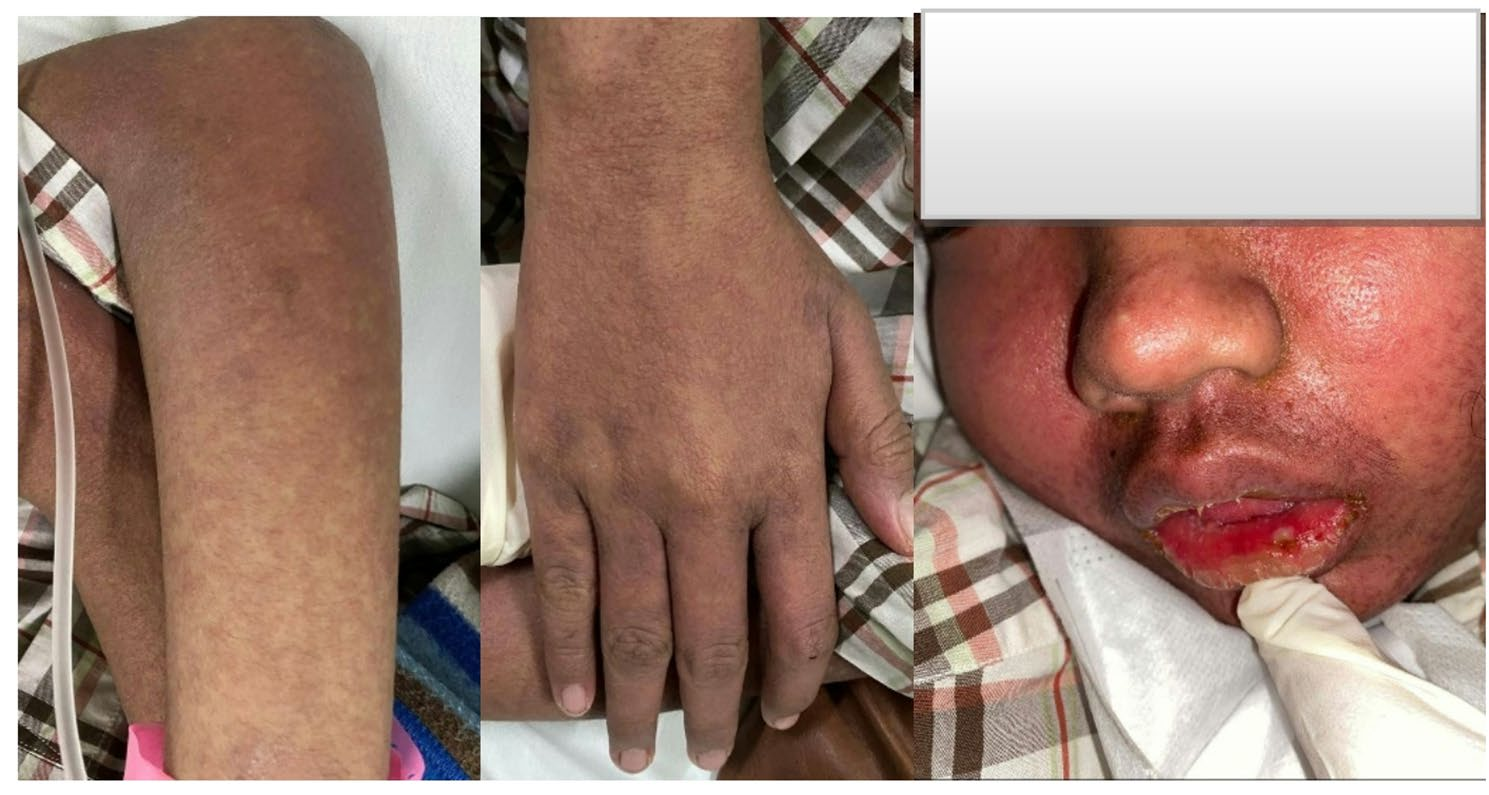
Face and Extremity of patient

The patient was readmitted with three days of watery diarrhea, occuring approximately 10 times/day, without blood or mucus. Associated symptoms included nausea, vomiting, and jaundice. A generalized pruritic maculopapular rash developed five days prior to admission, as detailed in Fig. 3. The rash was widespread, specifically on the face, neck, chest, abdomen, and upper limbs. Although submandibular lymph node remained palpable, it had decreased in size. Liver enzymes levels (SGOT/SGPT) had increased to four times the normal range. Sputum test of TB bacteria were negative, and chest X-ray showed improvement with no pleural effusion. The rash was diagnosed as a probable maculopapular drug eruption or DRESS (Drug Reaction with Eosinophilia and Systemic Symptoms), but eosinophilia was not present. Anti-TB drugs were temporarily discontinued and replaced with levofloxacin and ethambutol. Topical treatments included desoximetasone 0.25% cream, mometasone furoate 0.1%, and urea 10% lotion. At the 4-month follow-up, the skin rash persisted, appearing red-black and causing a burning sensation upon reintroduction of rifampicin. By the 5th month, respiratory symptoms, diarrheal, nausea, and vomiting had vanished, lymphadenopathy had decreased. However, the rash recurred consistently upon re-exposure to rifampicin, prompting its permanent discontinuation and the reintroduction of ethambutol. By the 6th month, all clinical symptoms were resolved, except for residual dark discoloration from the rash. During the 7-month follow-up at the haematology clinic, the skin rash had completely resolved, and no other complaints remained. Although injectable anti-TB therapy was proposed, it was declined by the patient’s family. At the 8-month follow-up, the patient remained symptom-free. A feeling of low self-confidence due to the syndrome was observed, and the father decided to discontinue medical therapy believing that the child had recovered and no longer needs further treatment.

## Discussion

**Fig. 4:**
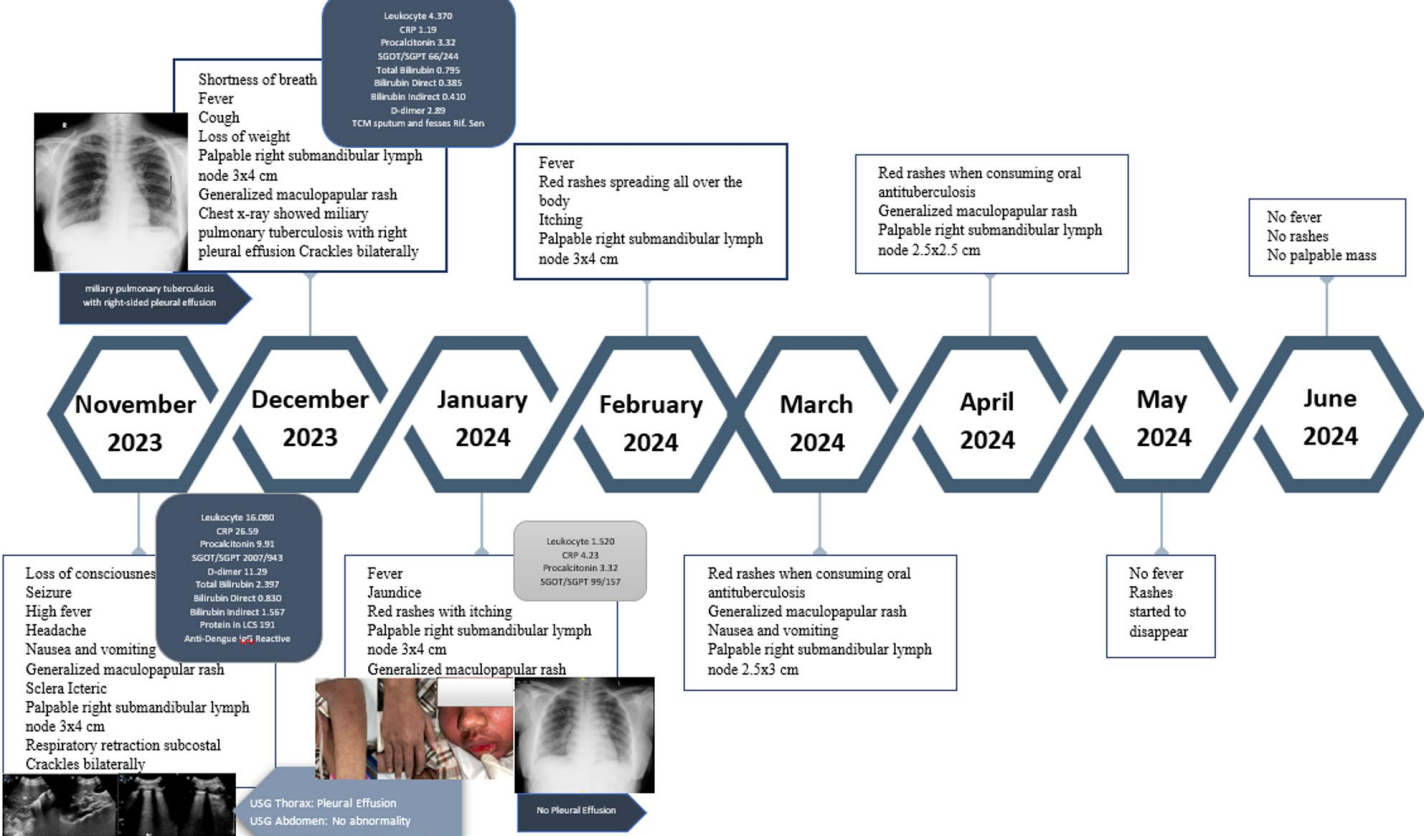
Course of Illness

MIS-C typically appears between 2 and 8 weeks after the peak of the infection. This indicate that the condition is post-infectious and mediated by immune system [18]. Similarly, the patient the present case developed MIS-C around 8–9 weeks after having a mild COVID-19 infection. The clinical manifestations are observed a few weeks after exposure, starting with general and non-specific symptoms. Fever is among the most common symptoms and considered as the cardinal sign of the syndrome, with a typical duration of 4 to 6 days. Gastrointestinal symptoms, including abdominal pain, vomiting, and diarrheal are observed in over 60% of cases. Non-specific skin rash also occurs in 45–75% of patients and in 30–75% cases, inflammation of the lips or tongue mucosa resembling Kawasaki Disease could appears. Ocular complication including non-purulent conjunctivitis occurs in 30–80%. Severe complications, including neurocognitive symptoms such as altered consciousness, seizures, headaches, lethargy, and irritability, occur in fewer than 50% of cases. Cardiology complication include arrhythmias or cardiovascular dysfunction. Respiratory symptoms are uncommon but often accompanied by other infections. Multisystem inflammation may also result in pleural effusion, pericardial effusion, or ascites [9]. In this case, the patient presented with fever, abdominal pain, vomiting, and general rash. Decreased consciousness was also observed, while severe symptoms were evidenced by persistent bradycardia and the imaging of pleural effusion (Fig. 4).

There are three primary diagnostic criteria used for identifying MIS-C. In this case, the diagnostic criteria were fully met based on both WHO and CDC definitions [8]. Fever lasting more than three days, elevated inflammatory markers, no identifiable microbial cause, evidence of prior COVID-19 infection, clinical features including rash, shock, coagulopathy (elevated PT, aPTT, INR, and D-dimer), and acute gastrointestinal symptoms (vomiting, abdominal pain, and diarrhea).

Management of MIS-C is divided into three main categories, the usage of intravenous immunoglobulin (IVIG), steroid, and symptomatic medication. IVIG and steroid can be used to modulate the immune response. Several studies have compared the usage, but the results remain uncertain [9, 18]. The recommended dose of IVIG is 2 g/kg/day once, while the dose of methylprednisolone varies from 2 to 10 mg/kg/day for 3 days [19]. In this case, the patient responded positively to corticosteroid therapy alone. Symptomatic therapies can also be used to reduce inflammation, including agents such as anakinra (an IL-1 receptor antagonist), tocilizumab, and infliximab (monoclonal antibodies targeting IL-6) [19]. Endothelial and cardiac dysfunction can lead to hypotension, hypoperfusion, and other organ dysfunction, making the use of vasoactive drugs necessary. Norepinephrine is typically administered in cases of vascular abnormalities, while epinephrine is preferred in the presence of cardiac dysfunction. As part of MIS-C protocols, anticoagulation with agents such as enoxaparin is often initiated until inflammation and D-dimer levels decreases [19]. In this case, the patient received intravenous methylprednisolone and responded well. Clinical and laboratory improvement was observed by the fifth day of therapy. Additionally, vasoactive agents such as epinephrine and norepinephrine were administered to treat circulatory failure. Anticoagulant enoxaparin was also administrated to manage hypercoagulability. Enoxaparin can be used in thrombocytopenic pediatric patients by adjusting the dose when the platelet counts are low [20, 21]. To monitor for the adverse effect and complications, the patient was observed for the sign of bleeding, thrombocytopenia, and renal function test. During admission, there were no sign of spontaneous bleeding, no decrease in platelet count and no increase in creatinine [22]. The patient responded well to the MIS-C treatment protocol and no longer required intensive care by the 11th day of hospitalization.

Patients with MIS-C experience excessive hyperactivation of the immune system, producing large amounts of pro-inflammatory cytokines such as interleukin-6 (IL-6), interleukin-1β (IL-1β), and tumor necrosis factor-alpha (TNF-α), a phenomenon known as a cytokine storm [9]. This condition leads to severe inflammation and damage to various organs in the body, including multi organ failure. The mechanism can interfere the body’s immune defense against invading pathogens, including *Mycobacterium tuberculosis* [16, 17]. Coinfection with SARS-CoV-2 and *Mycobacterium tuberculosis* disrupts the host’s immune response and worsens COVID-19 severity [16]. This may be due to an increase in circulating myeloid cell subpopulations that found in the lungs of patients with severe symptoms [18] and supports to the development of active or latent TB. The significant increase of interferon (IFN) production during COVID-19 [17] may trigger TB with poor outcomes. Furthermore, SARS-CoV-2 causes immunosuppressive effects that leads to the reactivation and dissemination of *Mycobacterium tuberculosis* infection [17]. Reduction in *Mycobacterium tuberculosis* and specific CD4 T cells in patients with COVID-19 may impair the ability to control both latent and new TB infections [18, 23]. In patients with MIS-C, an excessive immune response can disrupt granuloma integrity, enabling *Mycobacterium tuberculosis* to spread throughout the body, this condition referred as disseminated TB [17]. 

Children with latent TB are asymptomatic but have a hinger risk of reactivation, especially in countries with high TB prevalence like Indonesia [16]. Study showed that screening before starting immunosuppressive therapy is essential. Prophylactic anti-TB treatment should also be considered as part of the management of MIS-C. The primary treatment includes corticosteroid and IVIG. Corticosteroid suppress the production of pro inflammatory cytokines and reduce the immune system, while IVIG helps control overactive immune response. The treatment can weaken the immune system’s ability to fight other infections, such as TB. Managing coinfection of MIS-C and TB such a challenge for clinicians. Both conditions may present with similar clinical features, including fever, respiratory distress, and systemic inflammation, which emphasize the importance of early and accurate diagnosis. The use of immunosuppressive agents in MIS-C should be accompanied by anti-TB therapy to prevent the progression of the infection [16]. In this case, the patient showed symptoms of TB following a prior SARS-CoV-2 infection. Disseminated TB was confirmed bacteriologically through the detection of acid-fast bacilli (AFB) using TCM (GeneXpert) testing of sputum, urine, and fecal samples. Chest X-ray results further affirmed the presence of advanced pulmonary engagement consistent with miliary TB.

TB treatment includes a combination of several anti-TB drugs such as isoniazid, rifampicin, pyrazinamide, and ethambutol. Using multiple drugs at a time increases the risk of hypersensitivity reactions. These occur when the immune system mistakenly identifies the medications [24]. In MIS-C, the immune system becomes overactive, which can disrupt immune tolerance and make children more prone to develop hypersensitivity reactions. After the acute phase of the syndrome, some children experience weakened immune function and increasing risk of drug allergies. They may react to more than one TB drug due to factors such as cross-reactivity, specific genetic traits, or prolonged exposure that sensitizes the immune system [5]. In this study the patient showed a severe allergic reaction with skin manifestations classified as a cutaneous adverse drug reaction. The patient experienced itching, burning sensation on the skin, and generalized rashes shortly after taking oral anti-TB drugs. A more severe reaction had been observed before and recurred when the drug was re-administered. The symptoms improved after the drug was stopped, and no other potential causes were identified. Based on the Naranjo Adverse Drug Reaction Probability Scale, the score was 9, indicating a definitive adverse drug reaction and suggesting the possibility of Drug Reaction with Eosinophilia and Systemic Symptoms (DRESS).

The management of anti-TB drug allergy in a patient with disseminated TB and a history of MIS-C requires comprehensive clinical evaluation. Alternative regimens that could be tolerate by the patient can be adopted. Another option is implementing a desensitization protocol, where the medication is introduced gradually under strict medical supervision. In this patient, desensitization with first-line treatment had already been attempted, but allergic reactions still occurred. In cases of drug eruptions, both systemic and local treatment are necessary. In addition, family education plays a crucial role, particularly in emphasizing the risk of recurrence and the importance of treatment adherence, which support a good prognosis.

The limitations of this study include a focus on short-term management without assessment of MIS-C relapse, long-term tuberculosis control, or recurrence of hypersensitivity reactions. Furthermore, the investigation was conducted in a high prevalence country, and the results may not be fully applicable to lower TB burden area.

## Conclusion

In conclusion, the interaction between MIS-C, disseminated TB, and anti-TB drug allergies including anti TB drug-induced hepatitis (ADIH) represents a complex condition of the immune system dysregulation and hypersensitivity reactions. Immune dysregulation can damage multiple organs and increase susceptibility to disseminated TB. Additionally, this condition triggers hypersensitivity reactions to anti-TB drugs.

The complex interaction showed significant challenges in clinical management. A deep understanding of the relationship between MIS-C, disseminated TB, and drug hypersensitivity is essential for developing effective treatment strategies to optimize patient outcomes. Further study is needed to develop better diagnostic and therapeutic methods, as well as evaluate the effectiveness and safety of various desensitization protocols as well as alternative anti-TB drug regimens in patients with MIS-C. Better understanding of these interactions is essential for developing comprehensive, evidence-based guidelines to manage such complex conditions.

## Data Availability

The data used to support the findings of this study are included within the article.

## Abbreviations

ANA: Antinuclear Antibody
AFB: Acid-Fast Bacilli
CKM: Creatine Kinase–Myocardial Band
CMV: Cytomegalovirus
COVID-19: Coronavirus Disease 2019
CRP: C-Reactive Protein
D-dimer: D-dimer
EBV: Epstein–Barr Virus
Hb: Hemoglobin
HIV: Human Immunodeficiency Virus
Ht: Hematocrit
IgG: Immunoglobulin G
IgM: Immunoglobulin M
K: Potassium
MIS-C: Multisystem Inflammatory Syndrome in Children
Na: Sodium
PICU: Pediatric Intensive Care Unit
PT/aPTT/INR: Prothrombin Time / Activated Partial Thromboplastin Time / International Normalized Ratio
SGOT/SGPT: Serum Glutamic Oxaloacetic Transaminase (AST) / Serum Glutamic Pyruvic Transaminase (ALT)
TB: Tuberculosis
USG: Ultrasonography
Ur/Cr: Urea / Creatinine
